# In Vitro and In Vivo Biocompatibility Assessment of a Thermosensitive Injectable Chitosan-Based Hydrogel for Musculoskeletal Tissue Engineering

**DOI:** 10.3390/ijms241310446

**Published:** 2023-06-21

**Authors:** Barbara Canciani, Francesca Semeraro, Valentina Rafaela Herrera Millar, Francesca Gervaso, Alessandro Polini, Antonella Stanzione, Giuseppe Michele Peretti, Alessia Di Giancamillo, Laura Mangiavini

**Affiliations:** 1 IRCCS Ospedale Galeazzi-Sant’Ambrogio, Via Cristina Belgioioso 173, 20161 Milan, Italy; barbara.canciani@grupposandonato.it (B.C.); giuseppe.peretti@unimi.it (G.M.P.); 2 School of Medicine, Vita-Salute San Raffaele University, Via Olgettina 58, 20132 Milan, Italy; fra.semeraro1@gmail.com; 3 Department of Biomedical Sciences for Health, University of Milan, Via Mangiagalli 31, 20133 Milan, Italy; valentina.herrera@unimi.it (V.R.H.M.); alessia.digiancamillo@unimi.it (A.D.G.); 4 CNR NANOTEC—Institute of Nanotechnology, c/o Campus Ecotekne, Via Monteroni, 73100 Lecce, Italy; francesca.gervaso@nanotec.cnr.it (F.G.); alessandro.polini@nanotec.cnr.it (A.P.); antonella.stanzione@nanotec.cnr.it (A.S.)

**Keywords:** chitosan, hydrogel, injectable, thermosensitive, scaffold, tissue engineering

## Abstract

Musculoskeletal impairments, especially cartilage and meniscus lesions, are some of the major contributors to disabilities. Thus, novel tissue engineering strategies are being developed to overcome these issues. In this study, the aim was to investigate the biocompatibility, in vitro and in vivo, of a thermosensitive, injectable chitosan-based hydrogel loaded with three different primary mesenchymal stromal cells. The cell types were human adipose-derived mesenchymal stromal cells (hASCs), human bone marrow stem cells (hBMSCs), and neonatal porcine infrapatellar fat-derived cells (IFPCs). For the in vitro study, the cells were encapsulated in sol-phase hydrogel, and then, analyzed via live/dead assay at 1, 4, 7, and 14 days to compare their capacity to survive in the hydrogel. To assess biocompatibility in vivo, cellularized scaffolds were subcutaneously implanted in the dorsal pouches of nude mice and analyzed at 4 and 12 weeks. Our data showed that all the different cell types survived (the live cell percentages were between 60 and 80 at all time points in vitro) and proliferated in the hydrogel (from very few at 4 weeks to up to 30% at 12 weeks in vivo); moreover, the cell-laden hydrogels did not trigger an immune response in vivo. Hence, our hydrogel formulation showed a favorable profile in terms of safety and biocompatibility, and it may be applied in tissue engineering strategies for cartilage and meniscus repair.

## 1. Introduction

Musculoskeletal disorders, especially articular diseases such as meniscus and cartilage lesions, are some of the leading contributors to disabilities worldwide. Their impact on the non-fatal burden of diseases is trending upward due to the growth in the global aging population, corresponding to an increase in life expectancy and a decline in premature mortality rates. The repercussions of impairments in musculoskeletal health, epidemiologically measured as years lived with disability (YLDs), stand out in several aspects of individuals’ social and working lives, causing early retirement and reduced functional independence and capacity to actively engage in society [1,2,3]. Up-to-date approaches to cartilage and meniscus repair comprise the use of autografts or allografts. However, their drawbacks, including donor-site morbidity, a limited quantity of graft available for harvest, disease transmission, immune rejection, and alterations in natural extracellular matrix properties due to sterilization processes, make these treatments suboptimal for the overall patient population [4,5,6]. In light of these considerations, tissue engineering represents a promising strategy to improve the recovery from and results of cartilaginous and meniscal lesions, both as a stand-alone treatment and as an adjuvant to surgical care [7,8]. The successful design and production of a biological substitute aims to mimic the mechanical and functional characteristics of the native tissue and induce an adequate regenerative response. For this purpose, the accurate choice of cells, scaffolds, and signaling molecules is essential [9]. Mesenchymal stromal cells (MSCs) are the most promising cell source for tissue engineering in regenerative medicine applications due to their multipotency, high proliferative potential, and relative simplicity of isolation and growth [10,11,12,13]. Though cells are undoubtedly a crucial element of a tissue, a three-dimensional (3D) structure resembling the spatial organization and mechanics of the addressed tissue is needed to promote cell migration, proliferation, and differentiation. Among the 3D scaffolds, hydrogel systems constitute a valuable tool to harness a dynamic environment tunable to host cells. The careful selection and combination of the different polymers allow for the adjustment of hydrogel’s porosity, degradation, mechanical, and surface properties and, consequently, sets the most comparable surroundings to the in vivo conditions for encapsulated cells [14,15]. A significant advantage of hydrogels is their injectability, along with their biocompatibility and high water content, allowing the cell-laden scaffold to polymerize in situ and acquire the desired geometry in musculoskeletal tissue engineering.

Here, we investigated, in vitro and in vivo, the viability of different primary mesenchymal stromal cells in a thermosensitive, injectable, chitosan-based hydrogel. Chitosan (Ch) is a cationic polymer derived from crustacean shells that has good hydrophilicity, biocompatibility, and biodegradability [16,17,18,19]. Ch in association with a weak base, such as beta-glycerophosphate (βGP) functioning as a gelling agent, can form thermosensitive injectable hydrogels able to gel at a temperature range between 32 and 37 °C [20,21,22,23], thus having precise in situ applicability in vivo. In combination with βGP, we also enriched the hydrogel with sodium hydrogen carbonate (SHC) [24] to reinforce its mechanical properties and stability [20,21,22,25].

## 2. Results

### 2.1. Cell Encapsulation in Hydrogel In Vitro

We studied three distinct cell types cultured in vitro to assess their viability within the chitosan-based hydrogel: human adipose-derived mesenchymal stromal cells (hASCs), human bone marrow stem cells (hBMSCs), and neonatal porcine infrapatellar fat-derived cells (IFPCs, also known as Hoffa’s fat pad-derived cells). To obtain the hASCs, we seeded adipose tissue-derived stromal vascular fraction cells (AT-SVF) harvested from lipoaspirates. Together with the above-mentioned cell types, a subset of AT-SVFs were directly encapsulated into the hydrogel to assess their survival.

**Figure 1:**
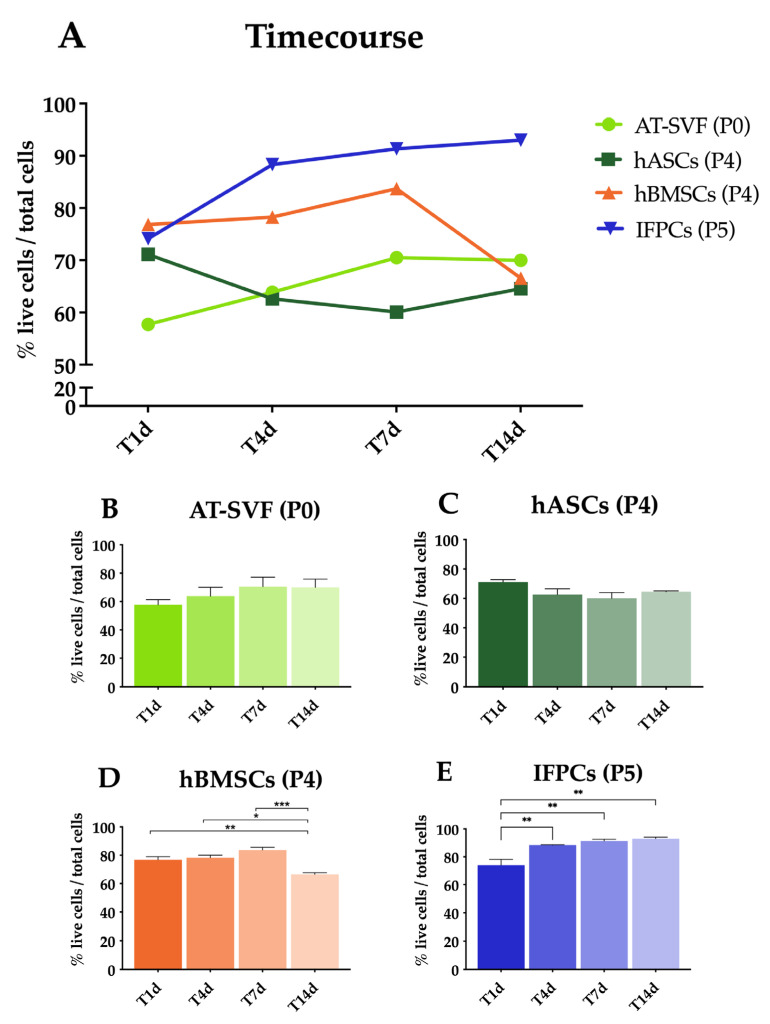
Chitosan-based hydrogel cell viability in vitro. (**A**) cell viability timecourse for stromal AT-SVF (light green line) encapsulated at P0, hASCs (dark green line) encapsulated at P4, hBMSCs (orange line) encapsulated at P4, and IFPCs (blue line) encapsulated at P5. (**B**–**E**) Statistic differences for each cell type during the timecourse (* *p* < 0.05, ** *p* < 0.01, *** *p* < 0.001).

The viability of all cell types was higher than 50% on all days in vitro (DIV, Figure 1A: timecourse). Considering each type of cell, hASCs (P0) were consistently viable throughout 14 DIV (Figure 1B), while hASCs (P4) showed a modest, non-significant decrement from 4 to up 14 DIV (Figure 1C). Conversely, hBMSCs displayed a slight increase at 7 DIV (Figure 1D), although a notable decline was evident at 14 DIV (Figure 1D). IFPCs exhibited a significant cell viability increase at 4 DIV (Figure 1E), persisting at 7 and 14 DIV (Figure 1E). Moreover, the viability of AT-SVF (P0) was significantly lower than hBMSCs, hASCs (P4), and IFPCs (Appendix A). This disparity was not evident at subsequent time points among human cells (Appendix A); however, it became more pronounced after 14 DIV in IFPCs compared with the other three cell types (Appendix A).

### 2.2. Hydrogel Ectopic Implant in Mice

#### 2.2.1. Histological Analysis

**Figure 2:**
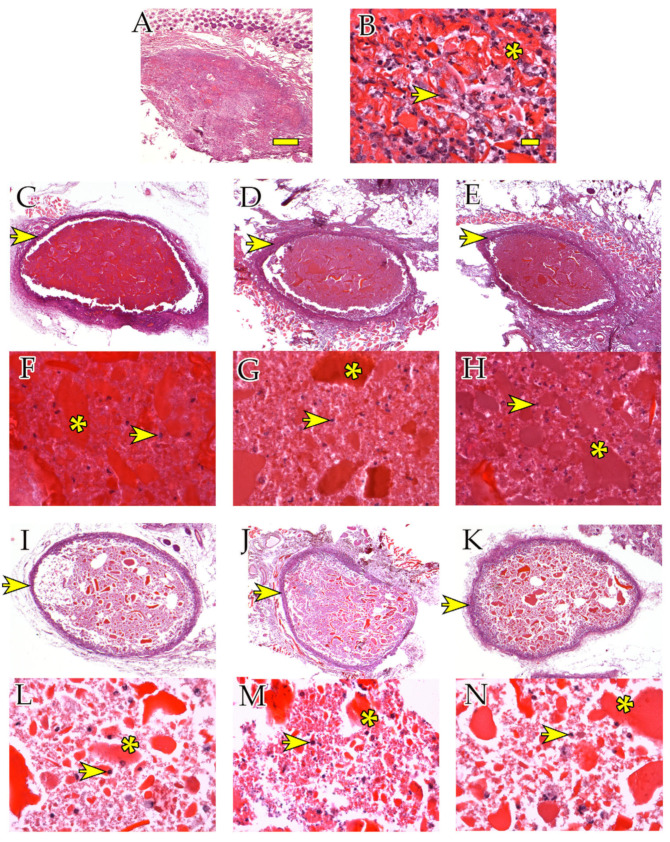
Scaffold morphology and presence of cells in hydrogels in vivo. H/E staining in hydrogel scaffolds harvested after 4 weeks (**A**–**H**) and 12 weeks (**I**–**N**). (**A**,**B**) Empty scaffolds (4 weeks) magnified at 2.5× and 40×, respectively. (**C**,**F**) hASC scaffolds (4 weeks) at 2.5× and 40×, respectively. (**D**,**G**) hBMSC scaffolds (4 weeks) at 2.5× and 40×, respectively. (**E**,**H**) IFPC scaffolds (4 weeks) at 2.5× and 40×, respectively. (**I**,**L**) hASC scaffolds (12 weeks) at 2.5× and 40×, respectively. (**J**,**M**) hBMSC scaffolds (12 weeks) at 2.5× and 40×, respectively. (**K,N**) IFPC scaffolds (12 weeks) at 2.5× and 40×, respectively. (**E**,**H**) IFPC scaffolds (12 weeks) at 2.5× and 40×, respectively. Asterisks point at denser hydrogel fragments. Arrows point at examples of cells in the hydrogel. Scale bars: (**A**) 100 μm, (**B**) 20 μm.

Hematoxylin/eosin staining was used for morphological evaluation of the subcutaneous hydrogel implants in athymic mice. The round-shaped scaffolds were consistent in size (from 3 to 5 mm in diameter) at both 4 and 12 weeks. They were clearly stained red-purple and were composed of denser fragments (asterisks in Figure 2) surrounded by a less compact matrix. After 12 weeks (Figure 2I–N), some degradation of the hydrogel was evident compared to 4 weeks (Figure 2C–H). The scaffolds displayed an external basophilic layer (arrows in Figure 2C–E,I–K) rich in cells and collagen. Such a layer was not present in empty scaffolds (Figure 2A, Appendix B). The cells were interspersed across the matrix (arrows in Figure 2B,F–H,L–N).

#### 2.2.2. Immunohistochemical Analyses

**Figure 3:**
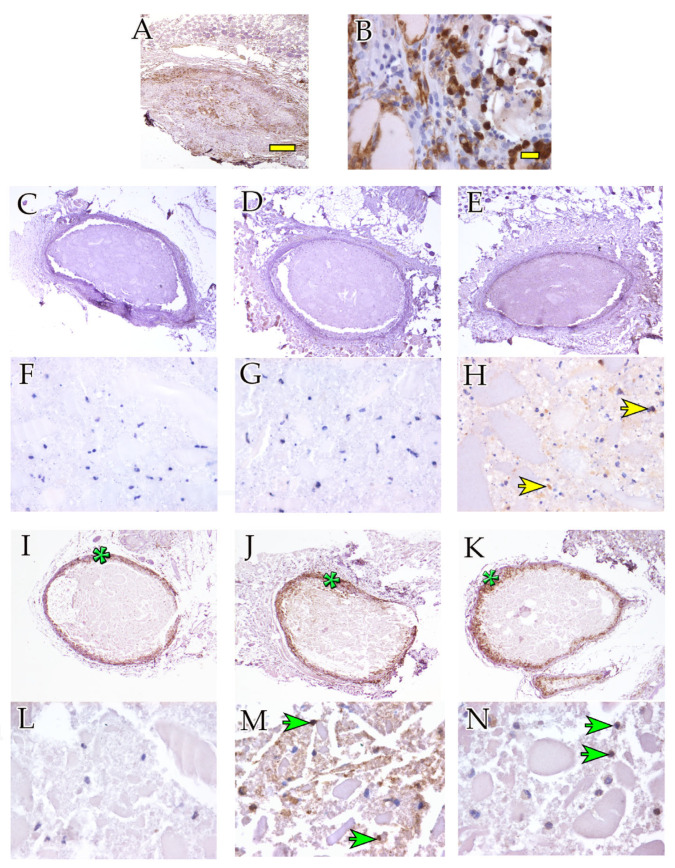
Macrophage-mediated inflammatory response after ectopic hydrogel implantation. Anti-F4/80 immunohistochemical staining showing hydrogel scaffolds harvested after 4 weeks (**A**–**H**) and 12 weeks (**I**–**N**). (**A**,**B**) Empty scaffolds (4 weeks) at 2.5× and 40×, respectively. (**C**,**F**) hASC scaffolds (4 weeks) at 2.5× and 40×, respectively. (**D**,**G**) hBMSC scaffolds (4 weeks) at 2.5× and 40×, respectively. (**E**,**H**) IFPC scaffolds (4 weeks) at 2.5× and 40×, respectively. (**I**,**L**) hASC scaffolds (12 weeks) at 2.5× and 40×, respectively. (**J**,**M**) hBMSC scaffolds (12 weeks) at 2.5× and 40×, respectively. (**K**,**N**) IFPC scaffolds (12 weeks) at 2.5× and 40×, respectively. (**E**,**H**) IFPC scaffolds (12 weeks) at 2.5× and 40×, respectively. Asterisks point at microphage-rich external layers of hydrogel scaffolds. Arrows point at examples of anti-F4/80-positive macrophages. Yellow arrows point at examples of anti-F4/80-positive cells at 4 weeks. Green arrows point at examples of anti-F4/80-positive cells at 12 weeks. Scale bars: (**A**) 100 μm, (**B**) 20 μm.

To distinguish between endogenous infiltrating cells (reacting to the ectopic implant) and surviving hASCs, hBMSCs, and IFPCs originally included in the hydrogels, we performed immunolabeling experiments using the anti-F4/80 antibody to specifically detect mouse macrophages. At 4 weeks, the hASC and hBMSC scaffolds were unstained (Figure 3C,D,F,G), while the IFPC scaffolds exhibited a few positive cells inside the hydrogel (Figure 3H, yellow arrows). After 12 weeks, positive cells were present around (Figure 3I–K, green asterisks) and inside (Figure 3M,N, green arrows) the hASC, hBMSC, and IFPC hydrogel scaffolds. Conversely, empty scaffolds were highly infiltrated by macrophages at 4 (Figure 3A,B) and at 12 weeks.

**Figure 4:**
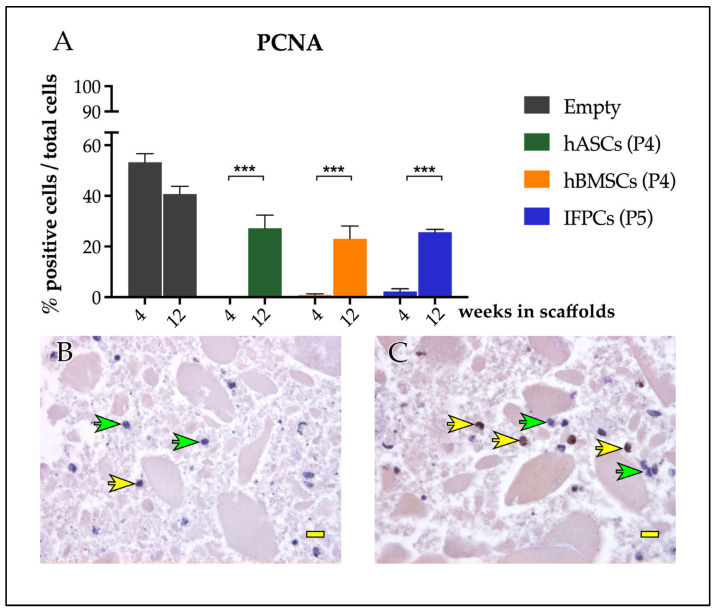
Cell proliferation. (**A**) Quantification of cell proliferation of cell-laden and empty hydrogel scaffolds. (**B**) Hydrogel scaffolds harvested after 4 weeks showed only a few PCNA-positive cells (yellow arrow) compared with negative cells (green arrows). (**C**) Hydrogel scaffolds harvested after 12 weeks showed an increased number of PCNA-positive cells (yellow arrows) compared with negative cells (green arrows). Statistic differences between 4 and 12 weeks (*** *p* < 0.001). Scale bars in (**B**,**C**): 20 μm.

Anti-proliferating cell nuclear antigen (PCNA) antibodies were used to evaluate cell turnover. The empty hydrogel showed PCNA positivity as early as 4 weeks, which remained constant up to 12 weeks. In contrast, hydrogels containing the other three cell types were negative for staining at 4 weeks, while antibody positivity significantly increased at 12 weeks (Figure 4).

## 3. Discussion

Notwithstanding the tremendous advances achieved in the field of regenerative medicine, the retrieval of damaged musculoskeletal tissues, especially cartilage and the meniscus, continues to pose a challenge. The basis of this shortcoming can be attributed to the lack of suitably engineered tissue matrices and scaffolds that can adequately replace the injured areas and facilitate tissue regeneration. Regarding the material prerequisites for regenerative medicine, the current focus on hydrogels is closely linked to their innate structural similarity to the extracellular matrix (ECM), and their ideal moisture retention property, porosity, biocompatibility, and biodegradable and biomimetic characteristics [26,27,28,29]. Thus, in this study, we decided to analyze a natural scaffold in in vitro and in vivo models. Our choice of a natural hydrogel to deliver a local cell-based therapy, instead of a synthetic system, was derived directly from both the above-mentioned characteristics and the well-known property of natural polymers to establish better interaction with living cells, given their ECM-like tissue composition [23,30,31]. The impact of hydrogels’ mechanical properties as tissue engineering scaffolds on encapsulated cells can be significant. It is a well-established fact that the ECM maintains isometric tension between cells within a specific tissue. Changes in these stresses can trigger a range of cellular responses, ranging from morphological alterations to modifications in gene expression at an individual cell level [32]. In this study, we utilized a combination of chitosan, βGP, and SHC to create a soft hydrogel [24]. Although its stiffness considerably differed from that of musculoskeletal tissues, previous research suggests that soft hydrogels are adequate to facilitate stromal cell commitment to chondrogenic and osteoblastic pathways [33,34,35,36]. Moreover, chitosan constitutes a promising option for musculoskeletal regeneration due to its plentiful availability, non-immunogenicity, antimicrobial and antioxidant properties, and natural biodegradability [17,18,19]. These characteristics could enhance the scaffold’s capacity to promote cell growth and facilitate the healing process.

In the present study, we investigated the in vitro viability of three cell lines loaded in an injectable chitosan-based hydrogel and its biocompatibility in an in vivo model. The Ch/βGP/SHC hydrogel exhibited strong support for the metabolism and growth of the encapsulated cells. Bone marrow-derived cells have been extensively characterized [37,38,39,40]; hence, the extraction method has attained widespread usage. Conversely, cells demonstrating multipotentiality derived from adipose tissue have garnered increased attention in the field of regenerative medicine in recent times [41,42]. The primary reason can be attributed to the relatively straightforward harvesting procedure and the widespread utilization of the stromal vascular fraction separation technique, which isolates cells with multipotentiality from the lipid fraction. Furthermore, the greater accessibility and the lower age-dependency of human lipoaspirate compared to bone marrow samples makes it a more convenient choice for application in regenerative medicine [43]. Therefore, for our study, we utilized cells extracted from lipoaspirate at passage 0 to explore the survival of these cells in the scaffold; they may be applied in one-step tissue engineering strategies in orthopedic practice, thus reducing site-donor morbidity and costs [44]. Additionally, we also employed cells extracted at passage 4, as the expansion process enables the selection of a greater number of cells through the culture method. We employed porcine infrapatellar fat-pad-derived cells as the third cell type, owing to the pig’s suitability as an experimental animal model. Consequently, an initial in vitro study was conducted to assess the viability of these cell types encapsulated in the hydrogel at various time points. Notable variations were observed between the experimental groups at 24 h (T1d) and 14 days (T14d) of culture. It is reasonable to suggest that the differences observed at 24 h may be attributed to both the cell type and passage, as at this time point, the percentage of live cells was significantly lower for the specific cell type under evaluation. While human adipose-derived stem cells (hASCs) were directly encapsulated at passage 0, the culture method applied to expand the other cell lines typically leads to a gradual selection of specific cell types from the extracted pool of cells. The three subsequent experimental intervals supported this hypothesis, since cell viability within the hydrogel ranged between 60 and 80% and displayed no substantial fluctuations between the experimental groups.

The present study also investigated the biocompatibility and safety of the chitosan-based hydrogel in a more complex environment. Ectopic cell-laden hydrogels of three specific cell types, namely hASCs (P4), hBMSCs (P4), and IFPCs (P5), along with a cell-free hydrogel, were implanted in athymic nude mice. The selection of these cell types at the specified passage was intended to standardize the procedure and ensure consistent cell density in each hydrogel implanted within each animal. Throughout the in vivo experiment, no indications of infection or rejection were observed in the mice, and the hydrogel remained subcutaneously intact. In addition, there were no observed signs of distress in the mice. Upon collecting the ectopic implant samples, a reduction in implant size was not noted over 12 weeks, while the internal degradation was attributed to the typical natural biodegradability process of the Ch-hydrogel [14].

The hydrogel scaffold supported the viability of encapsulated cells and enabled the infiltration of mouse macrophages and granulocytes both at 4 and 12 weeks in empty scaffolds. On the contrary, scaffolds enriched with human cells remained without mouse cell infiltration at 4 weeks. Collagen staining with picrosirius red stain revealed fibroblast activation, which formed the encapsulation layer around scaffolds enriched with human cells, but it was not evident in empty scaffolds. As expected, scaffolds enriched with human cells showed migration of the macrophage cells of the host only at the scaffold border at the longer time point of 12 weeks; however, the host cells did not deeply infiltrate the implanted substitutes.

The selected formulation of Ch-hydrogel employed in this study has not been previously documented for its application in musculoskeletal tissue engineering. Hoemann et al. [45] previously investigated a primary calf chondrocyte-loaded hydrogel composed of chitosan-based material. In their research, the hydrogel did not include SHC as a gelling agent, which enhances the mechanical properties of the hydrogel, as shown in other publications [20,21,22,25]. Furthermore, they investigated biocompatibility and musculoskeletal regenerative potential using differentiated cells at a substantially higher cell concentration compared to our study.

Considering the utilization of chondrocytes and the considerable cell quantity employed in their study, it would be challenging to translate their findings into clinical applications. This elucidates the rationale behind our exploration of the viability of human primary mesenchymal stromal cells from various sources, which have already been employed in musculoskeletal tissue engineering with different scaffolds. Establishing that the investigated formulation supports cellular metabolism and growth represents the initial crucial step toward considering the potential medical applications of such a hydrogel. Furthermore, we demonstrated that not only pre-expanded cells, which are more stable, but also freshly harvested cells (specifically, adipose tissue-derived stromal vascular fraction, AT-SVF), survive in this chitosan-based thermosensitive injectable hydrogel. This serves as the first evaluation of AT-SVF passage 0 cells in a chitosan-based thermosensitive injectable hydrogel.

Nevertheless, it is important to acknowledge the inherent limitations of this study that should be considered when interpreting the results. The influence of the intrinsic mechanical and biochemical properties of the scaffold on the mesenchymal cells’ commitment, differentiation, and capacity to contribute to the healing process in musculoskeletal regenerative medicine remains uncertain. Moreover, to achieve more accurate in vitro replication of the regenerative process, it would be ideal to incorporate additional environmental variables present in the in vivo setting, such as mechanical loading and hypoxia, both of which have demonstrated a fundamental role in the regenerative and homeostatic processes of various tissues (e.g., the meniscus) [46,47,48,49,50,51]. Thus, these variables will be more deeply investigated in future studies.

## 4. Materials and Methods

### 4.1. Chitosan-Based Hydrogel Preparation

The formulation of the hydrogel was obtained following the protocol presented in the literature [24]. Briefly, the hydrogel was prepared by mixing chitosan (Ch) with saline solutions of beta-glycerol phosphate (βGP) and sodium hydrogen carbonate (SHC). First, 3.33% Ch solution was prepared by dissolving Ch powder (deacetylation degree 75–85%, MW 50,000–190,000 Da, #448869, Sigma Aldrich, St. Louis, MO, USA) in 100 mL of 0.1 M HCl. The solution was left overnight under stirring at 20 °C until complete dissolution of the powder. The resulting solution was then centrifuged (2500 rpm, 5 min) to eliminate air bubbles. The gelling solutions (GA) of βGP (MW 306.11 g mol−1 #35675, Sigma Aldrich, St. Louis, MO, USA) and SHC (MW 84.007 g mol−1#401676, Sigma Aldrich, St. Louis, MO, USA) were prepared by dissolving βGP and SHC powders in Milli-Q water at initial concentrations of 0.5 M and 0.375 M, respectively. The hydrogel was freshly prepared by mixing Ch with two different gelling agent solutions (GAs), βGP, and SHC at 4 °C, with a respective ratio of 3:2 *v*/*v*, by introducing the GAs drop-by-drop into the chitosan to obtain a homogeneous and bubble-free solution.

### 4.2. In Vitro Study

We selected three different cell types to compare their capacity to survive in the hydrogel scaffold: 1. human adipose-derived mesenchymal stromal cells (hASCs), 2. human bone marrow stem cells (hBMSCs), and 3. neonatal porcine infrapatellar fat-derived cells (IFPCs, also known as Hoffa’s fat pad-derived cells).

**Figure 5:**
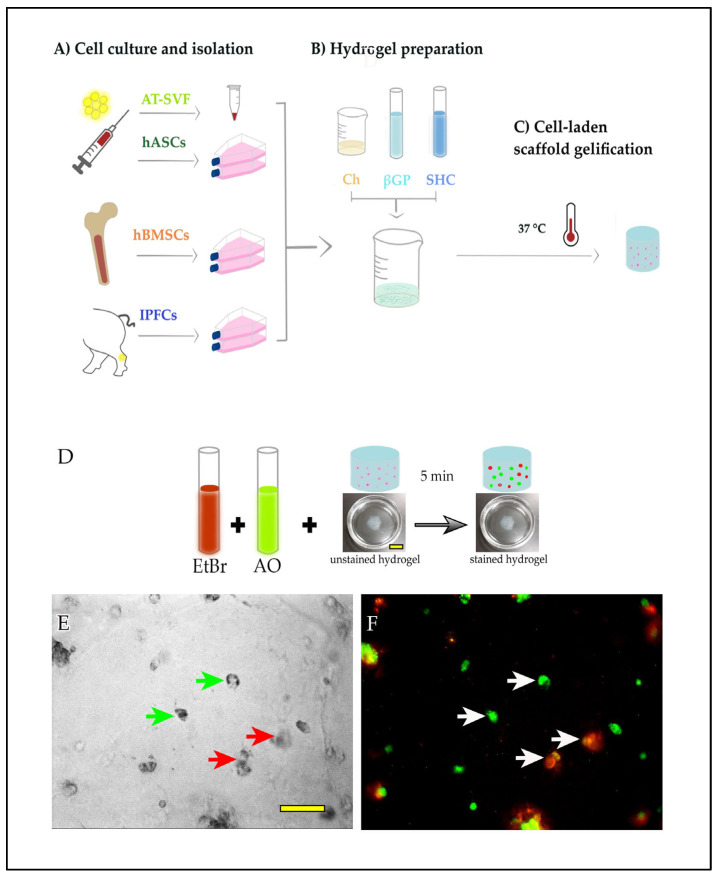
In vitro experimental design. (**A**) Three cell types selected to compare their capacity to survive in the hydrogel scaffold: adipose tissue-derived stromal vascular fraction (AT-SVF), human bone marrow stem cells (hBMSCs), and neonatal porcine infrapatellar fat-derived cells (IFPCs). (**B**) Hydrogel preparation. (**C**) Cell encapsulation and gelification of the hydrogel. (**D**) Live/dead assay protocol. EtBr: ethidium bromide; AO: acridine orange. (**E**) bright field microphotograph showing cells encapsulated in the hydrogel (green arrows point at examples of live cells; red arrows point at examples of dead cells). (**F**) Same image as E after fluorescent acquisition and color merge: live and dead cells were stained with AO (green) and EtBr (red), respectively. White arrows indicate live or dead cells. Scale bars: (**D**): 1 cm, (**E**,**F**): 50 μm.

hBMSCs were expanded up to passage 4, while IFPCs were expanded up to passage 5. These cells were subsequently encapsulated in the hydrogel (see below). A subset of adipose tissue-derived stromal vascular fraction cells (AT-SVF) were expanded up to passage 4 to obtain human adipose-derived mesenchymal stromal cells (hASCs), which were encapsulated in the hydrogel. Another subset of AT-SVF were directly encapsulated at passage 0. Cell preparation and hydrogel encapsulation procedures are summarized in Figure 5A–C.

Cells were encapsulated in the chitosan mixed solution, placed at 37 °C to gelatinize, and finally, cultured for up to 14 days (T14) within the specific medium for each cell type. The experimental time course started on the day of cell encapsulation. Cells in hydrogel were analyzed at 1, 4, 7, and 14 days after the day of cell encapsulation, and each time point was replicated three times.

#### 4.2.1. Isolation and Culture of Bone Marrow Stem Cells

Approval of the procedure was obtained from the Ethical Committee of IRCCS, Istituto Ortopedico Galeazzi (MS-TIP, approval number 214/INT/2020). Three patients (aged between 50 and 80 years old, body mass index (BMI) from 18 to 30 kg/m^2^) undergoing hip replacement procedures were selected. None of them had a history of diseases related to infection with HIV or HBV/HCV, bone metabolism, or joint inflammation.

Bone marrow aspirates from the femoral canal were collected and washed twice with 1× phosphate-buffered saline (PBS, Thermo Fisher Sci., Waltham, MA, USA) at 1700 g for 10 min at room temperature (RT). Nucleated cells were counted and plated at a density of 5 × 10^4^ cells/cm^2^ in alpha minimum essential medium (αMEM, Thermo Fisher Sci., Waltham, MA, USA), 10% volume/volume (*v*/*v*) fetal bovine serum (FBS, Euroclone, Milan, Italy), and 1% *v*/*v* penicillin/streptomycin/glutamine (P/S/G, Thermo Fisher Sci., Waltham, MA, USA), with 1 ng/mL recombinant human FGF-2 (Mylteni Biotec, Bergisch Gladbach, Germany). The primary cells were cultured at 37 °C and 5% CO_2_ for 1 week, and the medium was changed twice per week. The cells were expanded up to passage 4 (P4). We previously demonstrated that the hBMSC isolated following this protocol had the typical characteristics of mesenchymal stem cells (MSCs) because they were devoid of hematopoietic stem cells markers (CD34, CD45) and they expressed typical MSCs markers (CD73, CD90, and CD106). Furthermore, they were able to differentiate into osteocytes, adipocytes, and chondrocytes [37].

#### 4.2.2. Isolation and Culture of Human Adipose Stem Cells

Approval of the procedure was obtained from the Ethical Committee of IRCCS Ospedale San-Raffaele (MS-TIP, approval number 214/INT/2020)). AT-SVF were obtained from human adipose tissues of three patients, aged between 40 and 60 years old and undergoing knee injections for knee osteoarthritis (informed consent was given). None of them had a history of diseases related to infection with HIV or HBV/HCV. To obtain AT-SVF cells from lipoaspirate, a Hy-tissue SVF kit (Fidia Farmaceutici S.p.a., Abano Terme, PD, Italy) was used. This kit allows to harvest AT-SVF cells in a single surgical procedure. The lipoaspirate was homogenized, micro-filtered, centrifuged, and purified, according to Fidia’s protocol. AT-SVF from the three donors were encapsulated directly in the hydrogel. Moreover, an extra volume of AT-SVF obtained from donor 2 was seeded and incubated at 37 °C, 5% CO_2_ in Dulbecco’s modified Eagle’s medium/Nutrient Mixture F-12 (DMEM/F12, GIBCO, Thermo Fisher, Waltham, MA, USA) with 10% *v*/*v* fetal bovine serum (FBS, Euroclone, Milan, Italy). The medium was changed twice a week. The cells were expanded up to passage 4 (P4). These cells maintained the typical features of MSCs, as described in the literature [52].

#### 4.2.3. Isolation and Culture of Neonatal Porcine Hoffa’s Fat Pad Cells

Six samples of infrapatellar adipose tissue were harvested on a local farm from the knees of newborn piglet who had died under the weight of their mother (hence, no animals were sacrificed for experimental purposes). The tissues were cut into small pieces and digested in 5 mL of Dulbecco’s modified Eagle’s medium (DMEM, Thermo Fisher Sci., Waltham, MA, USA), 10% *v*/*v* fetal bovine serum (FBS, Euroclone, Milan, Italy), and 1% *v*/*v* penicillin/streptomycin/glutamine (P/S/G, Thermo Fisher Sci., Waltham, MA, USA) supplemented with 1 mg/mL collagenase I (Thermo Fisher Sci., Waltham, MA, USA) and 73.3% weight/weight (*w*/*w*) of CaCl_2_ (Sigma Aldrich, St. Louis, MO, USA) at 37 °C, and shaken continuously at 245 rpm for 1 h. To isolate the IFPCs, the digested tissue was filtered through a 70 µm nylon mesh to remove cellular debris, washed twice with equal volumes of PBS (1:1 PBS/digested suspension), and centrifuged at 500 g for 10 min. IFPC cells were cultured in DMEM (Thermo Fisher Sci., Waltham, MA, USA), 10% *v*/*v* FBS (Euroclone, Milan, Italy), and 1% *v*/*v* P/S/G (Thermo Fisher Sci., Waltham, MA, USA), and then, incubated at 37 °C and 5% CO_2_ and expanded up to passage 5 (P5).

### 4.3. Cell Encapsulation in Hydrogel

To produce cell-laden hydrogels, hASCs cells, hBMSCs, and IFPCs cells (3 × 10^6^ cells for each cell type) were detached using 0.5% trypsin-EDTA solution (Thermo Fisher Sci., Waltham, MA, USA) and centrifuged for 10 min at 500 g, and the pellet was resuspended in 50 μL of the medium. Fidia’s purification AT-SVF cells were centrifuged for 10 min at 500 g and the pellet was resuspended in 50 μL of medium. Each cell suspension was added to 1 mL of hydrogel formulation by gently mixing with a spatula to distribute the cells homogeneously. Drops (50 μL) of cell-laden hydrogels were distributed in 3.5 cm Petri dishes, and then, transferred at 37 °C to induce the sol–gel transition of the thermosensitive hydrogels. After 10 min, hydrogel spots were covered with cell-specific medium (2 mL), and incubated at 37 °C and 5% CO_2_.

### 4.4. Live/Dead Cells Assay

The live/dead assay was conducted to evaluate the viability of the primary cell lines in the hydrogels. Assays were performed using a staining solution composed of acridine orange (100 mg/mL, Sigma Aldrich, St. Louis, MO, USA) and ethidium bromide (500 μg/mL, Sigma Aldrich, St. Louis, MO, USA) freshly diluted to 1 μL/mL in PBS. The samples were washed with PBS and incubated within the staining solution at room temperature for three minutes in the dark. At the end of the incubation time, the staining solution was discarded and the hydrogel was covered with PBS. Images were acquired under fluorescence light microscopy (Olympus BX51WI, Shinjuku City, Japan), at 20× magnification with a water immersion objective. Acridine orange dye was excited at 480 nm, while the emission wavelength was set at 520 nm. Ethidium bromide was excited at 590 nm, while the emission wavelength was set at 640 nm. Each time point was replicated three times, with every repetition composed of 12 images to manually count viable, dead, and total cells. The results were expressed as percentages of the number of live cells over the total number of cells (Figure 5D–F).

### 4.5. In Vivo Study

Six female nude mice 086NU/Nu CD1 (52–56 days old), with a weight range of 25–30 g, were purchased from Charles River (Calco, Lecco, Italy). The animal study was conducted under the Ministry of Health license following the Animals (Scientific Procedures) Act C28/2022-PR. Animals were allowed to acclimatize for one week prior to experiments and housed in stable conditions (22 ± 2 °C) under a 12 h dark/light cycle with ad libitum access to food and water. The three primary cell types were encapsulated in hydrogels as described above. Briefly, hASCs (P0), hBMSCs (P4), and IFPCs (P5) were re-suspended in 50 μL of medium and added into 1 mL of sol-phase hydrogel; then, drops (50 μL) of cell-laden hydrogels were placed on round glass coverslips (diameter 13 mm). Subsequently, the coverslips were transferred at 37 °C to induce the sol–gel transition of the thermosensitive hydrogels. At the same time, empty hydrogels (i.e., devoid of cells) were obtained, following the same procedure as that for cell-laden hydrogels. Afterward, mice underwent general anesthesia, induced via intramuscular injection of a ketamine and xylazine mixture (Ketavet 100, MDS Animal Health S.r.l., Milan, Italy; Rompun 2% Bayer, Leverkusen, Germany; 0.5 μL/g and 0.25 μL/g, respectively).

**Figure 6:**
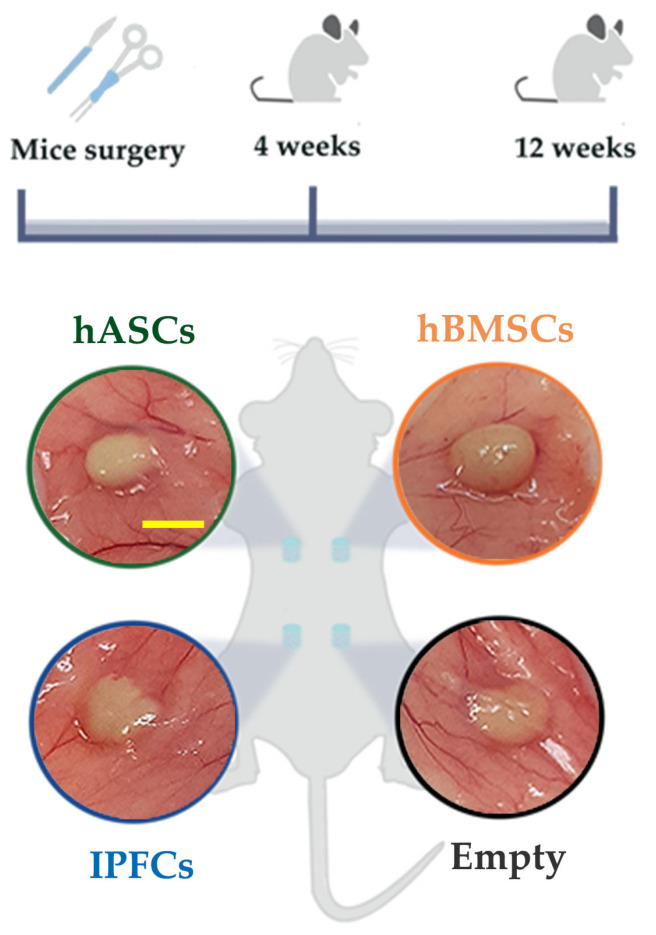
In vivo experimental design. Cell-laden and empty hydrogel scaffolds were inserted into four dorsal pouches in mice. Scaffolds were harvested after 4 and 12 weeks post-surgery. All scaffolds were evident after subcutaneous exposition. Scale bar for all images: 5 mm.

To obtain four dermal pouches of 1 cm length in each mouse, the dorsal skin was opened. Two anterior and two posterior dermal pouches were enlarged by using a blunt tweezer. hASCs, hBMSCs, IFPCs, and empty scaffolds were carefully pushed into the pockets using a spatula. The position of each type of scaffold in mice is reported in Figure 6. Next, the pouches were closed with absorbable 4.0 suture thread (Covidien Italia S.p.a, Segrate, MI, Italy). Postoperatively, animals were regularly monitored for signs of pain/infection, food intake, and activity during the entire experimental period. After 4 and 12 post-operation weeks, three mice for each time point were euthanized with an overdose of CO_2_, and the scaffolds were harvested (Figure 6). Scaffolds were processed for histology and immunohistochemistry for morphological analysis.

#### 4.5.1. Histological and Immunohistochemical Analyses

At the end of each time point, samples were fixed in 4% buffered formalin (Bio-Optica, Milan, Italy). After fixation, hydrogel implants were dehydrated in an increasing scale of ethanol (70%, 95%, and 100%, Carlo Erba, Milan, Italy), clarified in xylene (Bio-Optica, Milan, Italy) and embedded in paraffin (Bio-Optica, Milan, Italy). Finally, sections were cut using a microtome at a thickness of 5 µm. Hydrogel implant sections were de-waxed, and then, hydrated using xylene (Bio-Optica, Milan, Italy) and a decreasing scale of ethanol (100%, 95%, and 70%, Carlo Erba, Milan, Italy). For histology, hematoxylin/eosin (H/E; Bio-Optica, Milan, Italy) and picrosirius red (Abcam, Cambridge, UK) staining were carried out for morphological evaluation. For immunohistochemistry, sections were submerged in 3% *v*/*v* H_2_O_2_ for 30 min to block the endogenous peroxidase. Heat-induced antigen retrieval was performed in a citrate buffer (pH 6). Blocking of non-specific epitope binding was performed by incubating the sections in 10% *v*/*v* normal goat serum (NGS; Thermo Fisher Sci., Whaltham, MA, USA) in PBS for 1 h at room temperature (RT). The sections were incubated with the following primary antibodies diluted in 5% *v*/*v* NGS (Thermo Fisher Sci., Whaltham, MA, USA) in PBS, overnight, at 4 °C, in a humid chamber: anti-F4/80 (1:250 Cell Signaling Technology, Danvers, MS, USA) and anti-PCNA (1:10000 Abcam, Cambridge, UK). The sections were rinsed in PBS for 10 min three times, and then, incubated with goat anti-rabbit IgG biotinylated (Vector Laboratories Inc., Burlingame, CA, USA) and rat anti-mouse IgG1biotinylated (BD Bioscience, NJ, USA) antibodies diluted to 1:100 and 1:200, respectively, in 5% *v*/*v* NGS (Thermo Fisher Sci., Whaltham, MA, USA), in PBS, for 1 h, at RT. The sections were washed in PBS for 5 min three times, and subsequently incubated in an ABC Peroxidase staining kit (Thermo Fisher Sci., Waltham, MA, USA) for 1 h at RT. Finally, after rinsing in PBS for 5 min three times, 3,3′-diaminobenzidine (DAB, Sigma-Aldrich, St. Louis, MO, USA) was used as a chromogen reporter. The sections were counterstained with Harris hematoxylin (Bio-Optica, Milano, Italy) for a few seconds, subsequently dehydrated and cleared, and finally, sealed on coverslips using Eukitt mounting medium (Sigma-Aldrich, St. Louis, MO, USA). Images were acquired at 2.5× magnification using an Axiophot microscope (Zeiss, Baden, Wurttemberg, Germany) equipped with a Leica DFC450 C camera. Images were acquired at 40× magnification using a Leica DM5000 B microscope equipped with a Leica DFC480 camera (Leica, Wetzlar, Germany).

#### 4.5.2. Immunohistochemical Evaluation: PCNA Expression

PCNA expression was evaluated using a standard immunohistochemical procedure. A PCNA-positive cell was defined as one with clear, distinct brown nuclear staining. The number of positive cells was expressed as a percentage of the total number counted to generate a PCNA score. The final number of PCNA-positive cells was recorded from five images for each sample.

### 4.6. Statistical Analysis

Statistical analysis was performed using GraphPad Prism 8 Software. One-way ANOVA was used to compare the results, followed by Tukey’s post hoc test. Live/dead assay results and PCNA quantification were expressed as mean ± standard error of the mean (S.E.M.). Differences were considered significant at *p* < 0.05.

## Figures and Tables

**Figure A1:**
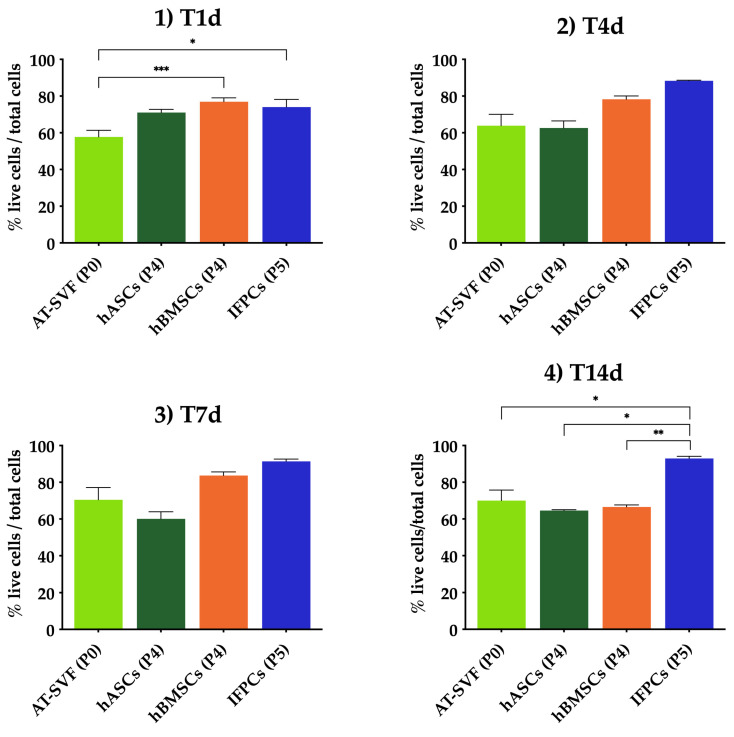
Chitosan-based hydrogel cell viability in vitro. (**1**–**4**) Cell viability of AT-SVF (light green column) encapsulated at P0, hASCs (dark green column) encapsulated at P4, hBMSCs (orange column) encapsulated at P4, and IFPCs (blue column) encapsulated at P5. Statistical differences for in vitro time point of each cell type (* *p* < 0.05, ** *p* < 0.01, *** *p* < 0.001).

**Figure A2:**
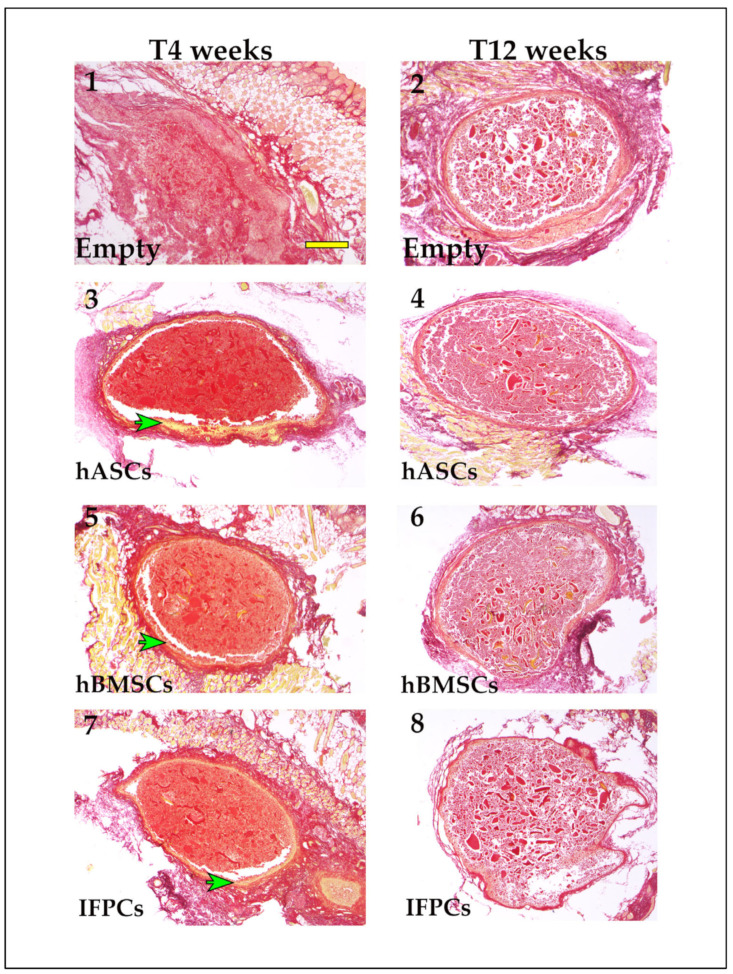
Picrosirius red staining to reveal collagen presence in hydrogel scaffolds. Scaffolds were collected after 4 (**1**,**3**,**5**,**7**) and 12 (**2**,**4**,**6**,**8**) weeks of implantation in mice. (**1**,**2**) Empty scaffolds, (**3**,**4**) hASCs scaffolds, (**5**,**6**) hBMSCs scaffolds, (**7**,**8**) IFPCs scaffolds. Green arrows indicate collagen in outer layers of scaffolds. Scale bar for all images: 100 μm.

## Data Availability

The data are available from the 26th of May 2023 at the following link: https://osf.io/b7kga/?view_only=18f0c890db87434d8d45db5d4e07ef37.
